# Healthcare utilization and cost of cancer-related care prior to allogeneic hematopoietic cell transplantation for hematologic malignancies in the US: a retrospective real-world analysis

**DOI:** 10.1186/s12913-021-07150-4

**Published:** 2021-10-20

**Authors:** Machaon Bonafede, Elias Anaissie, Kristin Evans, Robbin Itzler

**Affiliations:** 1 IBM Watson Health, Cambridge, MA USA; 2 CTI Clinical Trial and Consulting Services, Covington, KY USA; 3 CSL Behring, Marburg, Germany; 4 CSL Behring, King of Prussia, PA USA

**Keywords:** Hematologic malignancy, Hematopoietic cell transplantation, Claims-based study, Costs, Resource utilization

## Abstract

**Background:**

Hematopoietic cell transplantation (HCT) is a potentially curative therapy as well as a costly procedure. Published studies have examined the cost of HCT in the US and the complications that follow but little is known about the cancer-related healthcare costs and resource utilization prior to the procedure and none of the studies have examined the variability in cost based on the type of hematologic malignancy involved. The aim of this study was to estimate mean cancer-related costs and resources incurred before the HCT is performed from the time the hematologic malignancy first develops.

**Methods:**

The IBM® MarketScan® Research Databases were used to identify adult patients ≥18 years of age with commercial or Medicare supplemental insurance who had undergone allogeneic HCT for hematologic malignancies from January 1, 2008 to December 31, 2017. Healthcare utilization and costs were assessed during the 6 months prior to diagnosis (pre-diagnostic period) and the follow-up period from diagnosis just prior to the HCT (pre-HCT period). Multivariable regression models were constructed to estimate total all-cause costs and cancer-related costs as well as healthcare utilization by type in each time period.

**Results:**

A total of 2663 commercially insured patients and 266 with Medicare supplemental insurance were included in the study population. The mean-adjusted incremental cancer-related costs for commercially insured patients was $399,011 in the overall observation period including the pre-diagnostic and pre-HCT periods combined, 9% of which was incurred in the pre-diagnostic period. The corresponding mean-adjusted incremental cancer-related costs for Medicare supplemental patients was $195,575 for the same time period but the patterns of healthcare utilization were similar to the commercially insured population. Inpatient care accounted for approximately one-half the cost in both patient populations. By type of hematologic malignancy, costs were lowest for myeloproliferative disorders ($211,561) and highest for acute lymphocytic leukemia ($462,072) in the commercially insured population.

**Conclusion:**

This study demonstrates that overall patients with hematologic malignancies requiring HCT have considerable cancer-related healthcare resource utilization and costs leading up to HCT compared to the period of time prior to developing cancer.

**Supplementary Information:**

The online version contains supplementary material available at 10.1186/s12913-021-07150-4.

## Background

The use of hematopoietic cell transplantation (HCT) for the treatment of hematologic malignancies is steadily rising; it is a potentially curative and vital therapy for many patients but is associated with a significant cost burden [1–4]. Studies conducted in the US to date have focused on the resource utilization and costs from the time of the HCT procedure and a time period following the procedure. Two studies limited the follow up period to the first 100 days after the procedure [1, 2]. Two other studies followed patients for 1 year after the procedure [3, 4]. The high costs of HCT are partially related to post-HCT complications with previous studies showing higher healthcare costs in patients who developed complications compared with those who did not [4–6]. Only one study included cancer-related resource utilization and costs for a time period leading up to the HCT [3]. This study found that mean healthcare costs were $166,000 higher in the 6 months leading up to the procedure compared to control patients with hematologic malignancies who did not receive an HCT. For patients who underwent allogeneic HCT, total costs in the 6 months before and 12 months after the procedure were $377,958 higher than in control patients who did not undergo the procedure, with 82% of those costs being attributable to inpatient admissions and 15% to outpatient services [5].

Although the aforementioned study provided valuable information about costs incurred around the time of the HCT, the study period did not fully capture all cancer-related costs and healthcare utilization prior to the HCT. In addition, the patients with hematologic malignancies who undergo HCT differ from those patients who do not undergo HCT. For this reason, we chose to compare the costs of treatment before and after the cancer developed. To our knowledge, there is no existing literature describing cancer-related resource utilization for all time periods prior to the HCT.

The aim of this study was to estimate mean cancer-related healthcare resource utilization and costs for all time periods prior to the HCT including the tests and procedures required to diagnose the hematologic malignancy. The cancer-attributable healthcare resource utilization and costs were determined by comparing the utilization and costs before and after the hematologic malignancy among patients who received an allogeneic HCT. This was to minimize the risk of bias that would occur by comparing those receiving an allogeneic HCT with those who do not. The study also aimed to identify factors that influence these cancer-related costs. The quantification of the costs attributable to cancer with the current standard of care is very important in economic assessments. It allows health economists to determine whether introducing a new therapy to treat a hematologic malignancy may improve health outcomes and reduce healthcare utilization that will offset the cost of introducing a new therapy.

## Methods

### Data source

Adults with commercial or Medicare supplemental insurance with a healthcare claim for an allogeneic HCT and a prior diagnosis of a hematologic malignancy were identified in the IBM® MarketScan® Research Databases.

### Study population

Patients were included in the study if they had at least 18 months of continuous enrollment in MarketScan® prior to their hematologic malignancy diagnosis, with no evidence of a prior HCT. Patients who met the following inclusion criteria were selected for the study: 1) a healthcare claim with a procedure code for an allogeneic HCT from January 1, 2008 to December 31, 2017; 2) at least one non-diagnostic medical claim with a diagnosis of at least one of the hematologic malignancies listed below prior to the date of the claim for HCT, as early as July 1, 2005; 3) at least 18 years old on the index date (date of the first hematologic malignancy diagnosis) and; 4) at least 18 months of continuous medical and prescription coverage prior to the index date.

Diagnostic claims for the following hematologic malignancy diagnoses were included in the analysis: acute lymphocytic leukemia (ALL), acute myelogenous leukemia (AML), chronic lymphocytic leukemia (CLL), chronic myelogenous leukemia (CML), Hodgkin’s lymphoma, Non-Hodgkin’s lymphoma (NHL), multiple myeloma (MM), myelodysplastic syndromes (MDS), myeloproliferative disorders (MPD; including myelofibrosis, polycythemia vera and essential thrombocythemia), and aplastic anemia (AA).

Patients with the following criteria were excluded from the study sample: 1) any evidence of an allogeneic HCT prior to the index date; 2) ≥ 3 different hematologic malignancy diagnoses just prior to their HCT; 3) any of the following combinations of two different hematologic malignancy diagnoses just prior to their HCT: ALL + CLL, ALL + MM, AML + CLL, AML + Hodgkin’s lymphoma, AML + MM, CLL + CML, CLL + MM and CML + MM,; 4) patients with paroxysmal nocturnal hemoglobinuria were excluded due to the small sample size. We assumed those patients with three or more different hematologic malignancy diagnoses just prior to their HCT or those with combinations of two different hematologic malignancies are therapy-related diseases, where they are diagnosed with one hematologic malignancy and then develop another therapy-related hematologic malignancy as a result of treatment for the first hematologic malignancy diagnosed. These patients were excluded because we thought the costs of these patients would differ from most patients who were diagnosed with one type of hematologic malignancy. Forty patients were excluded for having three or more different diagnoses and an additional 45 were excluded for having one of the following combinations of two diagnoses: ALL + CLL; ALL + MM; AML + CLL; AML + Hodgkin’s; AML + MM; CLL + CML; CML + MM; CLL + MM.

### Study design

**Fig. 1:**
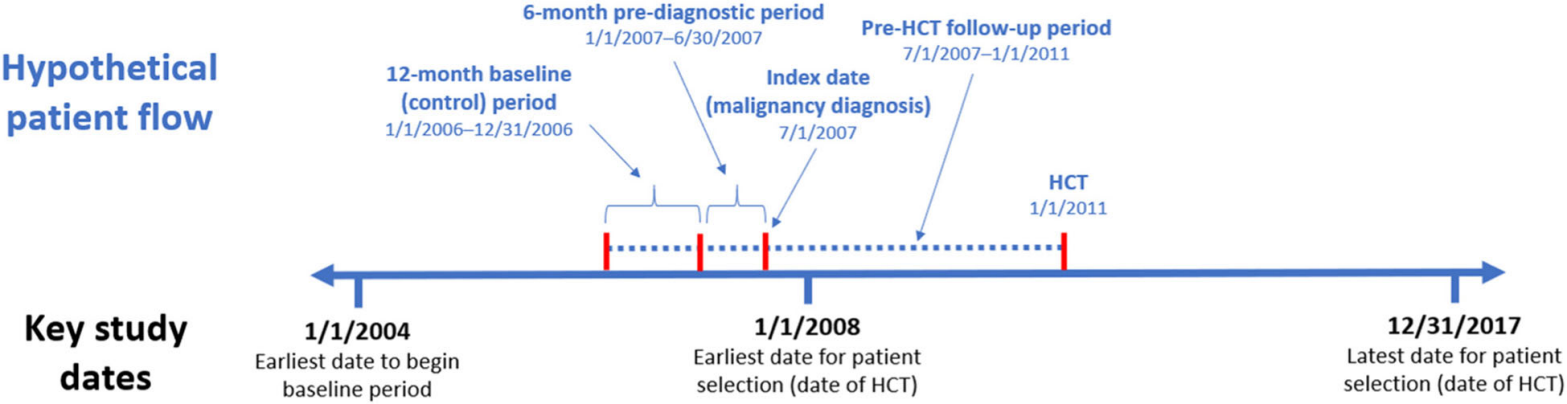
Hypothetical patient flow. Full study period (1/1/2004–12/31/2017) and analysis dates and time periods for a hypothetical patient who had HCT on January 1, 2011

The objective of the study was to compare the cancer-related healthcare resource utilization and costs once the individuals develop cancer with the time period before they develop their cancer. The 6 months prior to the first cancer diagnosis was included in the observation period in order to capture the costs of tests and procedures required to diagnose the cancer. Therefore, the time period from 18 months to 6 months before the hematologic malignancy diagnosis was considered the baseline/control period, the 6-month period prior to the hematologic malignancy diagnosis was considered the pre-diagnostic period, and the time from the hematologic malignancy diagnosis to the HCT was the pre-HCT follow-up period (Fig. 1).

Healthcare resource utilization (inpatient [IP] admissions, emergency room [ER] visits, outpatient [OP] services, OP prescriptions) and costs were quantified during the baseline, pre-diagnostic, and pre-HCT follow-up periods, and the overall pre-HCT period which was defined as the pre-diagnostic period (6 months before the first hematologic malignancy diagnosis) and the pre-HCT follow-up (period of time from first hematologic malignancy diagnosis up until the HCT is performed). Cancer patients undergoing HCT were not compared with matched controls without cancer as it would be difficult to control for all the potential differences between these two groups of patients through propensity score matching. To ensure that all cancer-related costs were included, these were defined as the difference between baseline costs and pre-diagnostic as well as pre-HCT follow-up costs rather than specific diagnosis codes on the claims. This approach is more comprehensive than relying on specific cancer diagnosis codes and cancer-related procedure codes because the immunosuppressive treatments that cancer patients receive often lead to infections and/or may interact with and exacerbate other comorbid conditions indirectly related to the cancer diagnoses. For the commercially insured population, separate healthcare resource utilization and cost analyses were conducted. In addition to analyzing the costs for all patients undergoing allogeneic HCTs regardless of type of hematologic malignancy, the healthcare resource utilization and cost for the five most common types of hematologic malignancies were analyzed separately. These analyses could not be performed in the Medicare supplemental population due to the small sample size.

Patients were classified into a malignancy cohort based on their hematologic malignancy diagnosis on the date of the HCT, or the closest date to the HCT where a hematologic malignancy diagnosis was observed on a claim.

### Statistical analysis

Descriptive statistics were generated for categorical variables as the count and percentage of patients in each category. Continuous variables were summarized by means, standard deviations and 95% confidence intervals of the means and medians.

Multivariable regression models were constructed to estimate total all-cause costs in each time period to derive the cancer-related costs as well as different types of healthcare utilization. All-cause costs in the baseline period were subtracted from all-cause costs in the pre-diagnostic, pre-HCT, and the overall observation periods to determine patients’ cancer-related costs in each of the time periods. Separate models were developed for the commercially insured and Medicare supplemental populations. Models for commercially insured patients controlled for type of hematologic malignancy, payer type, demographic characteristics (age, sex, geographic region, insurance plan coverage, year of diagnosis), clinical characteristics (comorbid disease and medication use throughout the study) and time to HCT. For the Medicare models, only patient demographics and time to HCT were included due to the small sample size. Adjusted cancer-related healthcare utilization by type of service was modelled only for the commercially insured population because the sample size for the Medicare supplemental population was deemed too small.

Distribution of the outcome determined the statistical approach. Separate piecewise gamma family generalized linear models (GLM) with a log link were used to estimate the cancer-related costs for the overall pre-HCT period. These models employed smoothed penalized splines by diagnosis group to address heterogeneity in costs by hematologic malignancy type. Subset analysis was conducted to evaluate costs by hematologic malignancy. Hurdle models were used to estimate hospitalization and ER visit costs. Covariate-adjusted counts of IP hospitalizations and ER visits were estimated using negative binomial GLM models, while hurdle models were used to estimate the count of OP visits for the commercially insured population. When models did not converge, a hurdle model with a Poisson distribution was used.

All costs are expressed in 2018 constant US dollars, adjusted using the Medical Care component of the Consumer Price Index (CPI).

## Results

In total there were 9468 patients in the MarketScan® commercial and Medicare Supplemental Databases with a code for an allogeneic HCT within the study period; 2929 met all inclusion/exclusion criteria and were included in the study (commercially insured: *N* = 2663; Medicare supplemental: *N* = 266). Patients’ baseline characteristics are shown in Supplementary Table 1 (commercial) and 2 (Medicare supplemental). Sixty-four percent (*n* = 4185) of those excluded did not have ≥18 months of observation time prior to their hematologic malignancy diagnosis. Another 23.2% (*n* = 1518) did not have ≥1 diagnostic medical claim with a diagnosis for a hematologic malignancy. The remaining 12.8% (*n* = 836) were either not ≥18 years of age, had ≥ 2 different hematologic malignancy diagnoses just prior to their HCT, or had PNH, which was excluded from further analysis due to the small sample size.

Eighty-five percent of patients included in the study had at least one of the five main malignancy types: AML, NHL, MDS, ALL and MPD. The remaining 15% of the population had CLL, HL, MM, or AA.

Overall, results for the Medicare supplemental population were comparable to those for the commercially insured population. As most patients included in the analysis were commercially insured, results are presented for the commercially insured population only unless otherwise stated.

### Time from hematologic malignancy diagnosis to HCT

The mean (standard deviation [SD]) time from hematologic malignancy diagnosis to HCT was 12.3 (15.4) months for all patients, 12.5 (15.7) months for commercially insured patients and 10.5 (12.0) months for Medicare patients. The median time (range) from diagnosis to HCT was 6.3 (0.0–131.1), 6.3 (0.0–131.1) and 6.2 (0.1–85.3) months for all patients, commercially insured and Medicare patients, respectively.

Among commercially insured patients, 48.3% had their HCT within 6 months of their initial hematologic malignancy diagnosis, 21.5% between 6 and 12 months after diagnosis and 30.2% > 12 months after diagnosis; among Medicare supplemental patients, 48.8% had their HCT within 6 months after their initial hematologic malignancy diagnosis, 25.6% 6–12 months after diagnosis and 25.6% > 12 months after their initial diagnosis.

The mean time from hematologic malignancy diagnosis to HCT for commercially insured patients varied by the type of hematologic malignancy involved ranging from 8.1 months among ALL and AML patients, 12.4 and 14.5 months for MDS and MPD, respectively, and up to 21.0 months among NHL patients. Of ALL and AML patients, ≥60% received their HCT within 6 months of diagnosis while only 21.7% of NHL patients received their HCT within 6 months. (Table 1). The results were relatively similar for Medicare supplemental patients (data not shown).

**Table 1:** Time from initial hematologic malignancy diagnosis to HCT by type of hematologic malignancy. All commercially insured patients included. HCT, hematopoietic cell transplantation; SD, standard deviation

Diagnosis	Months to HCT		Proportion of patients with ≤ 6, > 6 and < 12, and ≥ 12 months between their initial diagnosis and HCT		
	Mean (SD)	Median	≤6 months	> 6 and < 12 months	≥12 months
All patients (*N* = 2663)	12.5 (15.7)	6.3	1287 (48.3%)	573 (21.5%)	807 (30.2%)
Acute lymphocytic leukemia (*N* = 346)	8.1 (10.0)	4.9	210 (60.7%)	82 (23.7%)	54 (15.6%)
Acute myelogenous leukemia (*N* = 984)	8.1 (10.5)	4.4	650 (66.1%)	156 (15.9%)	178 (18.0%)
Non-Hodgkin’s lymphoma (*N* = 447)	21.0 (20.8)	13.7	97 (21.7%)	105 (23.5%)	245 (54.8%)
Myelodysplastic syndrome (*N* = 330)	12.4 (17.4)	6.4	154 (46.7%)	90 (27.3%)	86 (26.0%)
Myeloproliferative disorders (*N* = 148)	14.5 (18.6)	7.8	61 (41.2%)	36 (24.3%)	51 (34.5%)

### Total cancer-related healthcare costs

**Fig. 2:**
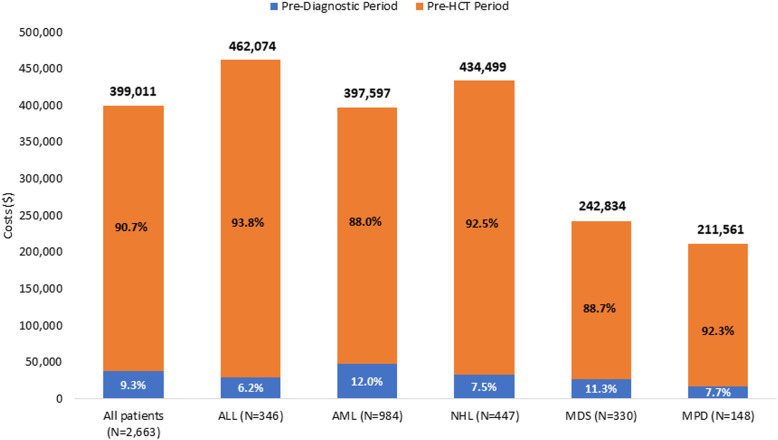
Adjusted mean cancer-related healthcare costs for the overall observation period by type of hematologic malignancy. All commercially insured patients included. Adjusted for patient demographic and clinical characteristics. The percentages shown are approximated using data from different analyses. ALL, acute lymphocytic leukemia; AML, acute myelogenous leukemia; HCT, hematopoietic cell transplantation; MDS, myelodysplastic syndrome; MPD, myeloproliferative disorders; NHL, Non-Hodgkin’s lymphoma

The mean-adjusted incremental cancer-related cost for commercially insured patients was $399,011 for the overall observation period including the pre-diagnostic and the pre-HCT periods combined. Approximately 9% of these incremental costs were incurred in the pre-diagnostic period, and the remainder were incurred in the subsequent pre-HCT follow-up period (i.e., the period from hematologic malignancy diagnosis up to HCT) (Fig. 2). The corresponding adjusted incremental cancer-related costs for Medicare supplemental patients was substantially lower at $195,575 for the pre-diagnostic and pre-HCT periods combined.

**Fig. 3:**
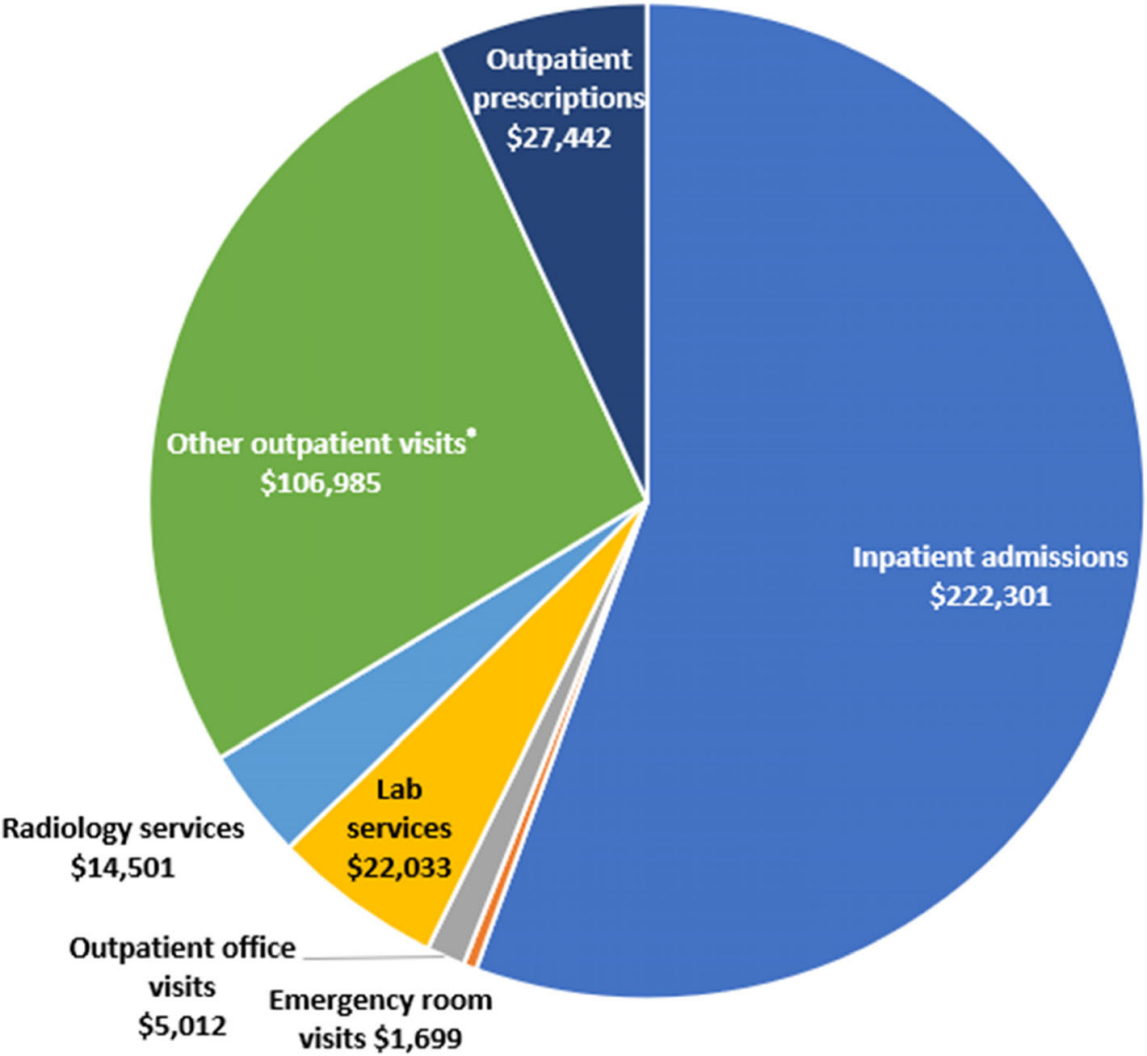
Adjusted mean cancer-related healthcare costs, overall and by service type, during the overall observation period. All commercially insured patients included. Adjusted using the Medical Care component of the Consumer Price Index. Note that there are slight differences in the overall mean cost regardless of hematologic malignancy and the sum of the mean costs by service type due to the use of different modelling techniques to fit the data. *Other outpatient visits include interactions not captured by office visits including home healthcare, walk-in health clinic services, and ambulatory surgical centers. HCT, hematopoietic cell transplantation

The mean-adjusted cancer-related healthcare costs during the overall observation period among commercially insured patients by hematologic malignancy diagnosis are shown in Fig. 2. Mean cancer-related costs varied among the five main hematologic malignancies of interest, ranging from $211,561 for MPD patients up to $462,074 among patients with ALL for the overall observation period. As shown in Fig. 3, inpatient costs accounted for approximately half of the mean cancer-related costs in the overall observation period among the commercially insured population. OP interactions accounted for 28% of the cancer-related costs and radiology services, lab services, and ER visits accounted for the remainder of the costs.

### Cancer-related healthcare utilization

The adjusted number of cancer-related hospitalizations, ER visits, and OP visits by time period among all commercially insured patients are shown in Table 2. Eighty-seven percent of commercially insured patients had at least one cancer-related hospitalization during the overall observation period, with an adjusted mean of 3.6 cancer-related hospitalizations. Fifty-six percent had at least one cancer-related ER visit (adjusted mean: 1.7 visits), and 96% had at least one cancer-related OP office visit (adjusted mean: 27.1 visits).

**Table 2:** Adjusted number of cancer-related hospitalizations, ER visits, and outpatient office visits by defined period. *OP office visits include only office visits and no other outpatient services. ER, emergency room; HCT, hematopoietic cell transplantation; OP, outpatient

	Commercially Insured Patients ***N*** = 2663		
Type of Cancer-Related Healthcare Interaction	Pre-Diagnostic Period	Pre-HCT Follow-Up Period	Overall Observation Period
≥ **1 cancer-related hospitalization (N, %)**	690 (25.9%)	2170 (81.5%)	2311 (86.8%)
Number of hospitalizations	0.43	3.21	3.61
≥ **1 cancer-related emergency room visit (N, %)**	678 (25.5%)	1227 (46.1%)	1503 (56.4%)
Number of emergency room visits	0.39	1.32	1.66
≥ 1 cancer-related OP office visit* (N, %)	2033 (76.3%)	2550 (95.8%)	2550 (95.8%)
Number of OP office visits	6.69	30.53	27.10

Separate healthcare utilization and cost multivariable models were developed for different hematologic malignancies among commercially insured patients so that this information would be available to researchers developing economic evaluations for different purposes. Although the differences in cancer-related costs for different hematologic malignancies were not tested for statistical significance, we did observe that there were differences between the healthcare utilization, cancer-related costs and timing of care across the five major hematologic malignancies. Among the five main diagnoses of interest, commercially insured ALL patients had the highest adjusted number of cancer-related hospitalizations (5.6) during the overall observation period, and MPD patients had the lowest (1.6); adjusted results were estimated using GLM including hematologic diagnoses, controlling for baseline hospitalizations (Table 3). The adjusted mean number of cancer-related ER visits was highest for commercially insured ALL patients (2.1) and lowest among AML patients (1.6). The adjusted mean number of cancer-related OP office visits ranged from 17.1 among AML patients, up to 23.3 among NHL patients.

**Table 3:** Adjusted mean number of cancer-related hospitalizations, ER visits, and outpatient office visits by diagnosis. Overall observation period used stratified by the five main hematologic diagnoses of interest. ER, emergency room

	Hospitalizations	Emergency room visits	Outpatient office visits
Acute lymphocytic leukemia (*N* = 346)	5.62	2.14	18.30
Acute myelogenous leukemia (*N* = 984)	4.26	1.55	17.10
Non-Hodgkin’s lymphoma (*N* = 447)	4.35	2.05	23.26
Myelodysplastic syndrome (*N* = 330)	2.00	1.74	20.18
Myeloproliferative disorders (*N* = 148)	1.62	1.75	19.67

Highest total cancer-related costs were observed for ALL and NHL ($462,074 and $434,499, respectively); costs for AML were somewhat lower ($397,597) while MDS and MPD were associated with relatively lower total cancer-related costs ($242,834 and $211,561, respectively) in the overall observation period. The higher costs among patients with NHL is best explained by the fact that NHL patients undergo several therapies before HCT including stem cell mobilization and autologous HCT, in the event of relapse following several cycles of chemotherapy, and the course of the disease leading up to the need for an allogeneic HCT usually spans two to three years [7]. Indeed, NHL had the slowest progression to HCT with 21.7% progressing within 6 months while the other malignancies had 40–60% progressing to HCT within 6 months. The costs of NHL stand in sharp contrast to the costs for MDS and MPD, which are typically more indolent conditions, usually managed with relatively inexpensive oral or subcutaneous agents before progressing, in a relatively small proportion of patients, to require allogeneic HCT (Tables 1 and 3). This is also reflected by the lower likelihood of hospitalization among patients with MDS and MPD (Table 3). The higher intensity treatments typically given to treat ALL and AML also explain the higher costs incurred over a shorter period before HCT.

Notably, the cost of treatment per month until HCT was approximately $40,000 for the entire study population and was highest for ALL and AML, approximately $57,000 and $49,000 per month respectively, and lowest for NHL, MDS and MPN ($21,000, $19,000, and $15,000 per month, respectively).

The results of multivariable analyses for total cancer-related costs during the diagnostic/pre-HCT period showed 13% greater adjusted costs for patients with their index diagnosis during 2010–2013 or during 2014–2017, compared to 2005–2009 (*p* < 0.05 for both).

## Discussion

In a retrospective claims-based analysis of 2663 patients with commercial insurance and 266 patients with Medicare supplemental insurance, mean cancer-related healthcare costs during the overall observation period were estimated at nearly $400,000 for commercially insured patients, and nearly $200,000 for Medicare supplemental patients, with nearly half of those costs incurred due to hospitalizations. Between 6 and 13% (depending on type of hematologic malignancy) of the costs were in the pre-diagnostic period.

The patterns of resource utilization for the Medicare supplemental population were similar to the commercially insured population but the costs of care were lower due to lower rates of Medicare reimbursement. We also observed differences in healthcare utilization and cost between the different hematologic malignancies as well as the timing of care across the five major hematologic malignancies. Highest total cancer-related costs were observed for ALL and NHL ($462,074 and $434,499, respectively); cancer-related costs for AML were somewhat lower ($397,597) while MDS and MPD were associated with relatively lower total cancer-related costs ($242,834 and $211,561, respectively) in the overall observation period. The differences in healthcare utilization and cost between the different hematologic malignancies are likely due to differences in the management of each malignancy and the position of allogeneic HCT in the therapeutic paradigm prior to HCT as well as the clinical course of each malignancy including mortality rates, health outcomes, and speed of progression. The distribution of costs did not necessarily correlate with the length of time from diagnosis of the hematologic malignancy to the HCT procedure because of the factors mentioned above including the course (indolent versus rapid) and the type of therapies used for the various underlying diseases. The time from hematologic malignancy diagnosis to HCT varied from around 8 months for ALL and AML, around one year for MDS and MPD to almost two years for NHL.

A significant effect of more recent diagnosis (during 2010–2013 or 2014–2017 vs. 2005–2009) on cancer-related costs is likely related to the novel therapies that became available, particularly for ALL during these separate study periods as well as the introduction of new diagnostics [8, 9]. It is difficult to include all the advances in treatments that contributed to the changes in cost over time; however, we adjusted costs using the medical component of the CPI, which should help stabilize costs across eras.

Prior research focused on the high costs associated with HCT and its subsequent complications [4–6]. Only one study examined the cancer-related costs in the six months prior to the HCT [3]. The results of this study are consistent with the earlier research, but the cancer-related costs reported prior to the HCT period are more comprehensive. Although the costs may differ depending on the type of underlying hematologic malignancy identified, the cancer-related costs are substantial for all types of hematologic malignancies. This earlier time period is vital to understanding the full breadth of HCT-related healthcare resource utilization and costs.

The limitations of this study include those inherent to retrospective claims-based analyses. Detailed medical histories are not available so the variables that can be evaluated are limited. Also, the sample with Medicare supplemental insurance was small. As a result, many of the analyses are limited to only those individuals with commercial health coverage. Therefore, the results may not be generalizable to patients with other insurance or without health insurance. However, it is important to note that the patterns of healthcare utilization for the Medicare population were similar even though the rates of reimbursement were different. Moreover, there is a potential for misclassification of hematologic malignancies, allogeneic HCT or other variables and outcomes. The healthcare utilization and costs for new comorbid conditions after baseline were not identified separately but they were controlled for in the multivariable models. It is difficult to distinguish between conditions unrelated to the cancer diagnosis and those indirectly caused by the diagnosis of the hematologic malignancies. Lack of a diagnosis on a claim in the pre-index period does not necessarily mean the patient did not have the condition during baseline. Often after receiving a serious diagnosis such as cancer, a patient may have more frequent healthcare visits, and therefore pre-existing conditions may be identified as they are being diagnosed with a hematologic malignancy. Also, patients diagnosed with hematologic malignancies may require more medical resources for other comorbid conditions because of their cancer diagnosis and the fact that they are immunosuppressed. Despite these limitations, the majority of the patients undergoing HCT are commercially insured, the sample size is large and the time from onset of the hematologic malignancy to the HCT procedure is short so it is unlikely that the increase in comorbid conditions would have occurred in the absence of the cancer diagnosis. Finally, the costs for those who undergo HCT should not be compared to costs of those who do not undergo an HCT because these two groups of patients are not comparable with respect to their health status.

## Conclusions

This analysis quantifies the cancer-related resources and costs incurred from the time patients develop their hematologic malignancy to the time they undergo an HCT. This analysis demonstrates that the cancer-related resources incurred from the time patients develop their hematologic malignancy to the time of the HCT are substantially and significantly higher than baseline costs for patients who progress to HCT. The cost estimates derived from these types of studies provide important information for decision making from an economic perspective.

## Supplementary Information

**Additional file 1.**

## Data Availability

The datasets analyzed during the current study are not publicly available due to data use agreements with the underlying data contributors that restrict access to the data and prohibit the data from being publicly available. Data are available from the corresponding author on reasonable request.

## Abbreviations

AA: aplastic anemia
ALL: acute lymphocytic leukemia
AML: acute myelogenous leukemia
CLL: chronic lymphocytic leukemia
CML: chronic myelogenous leukemia
ER: emergency room
GLM: generalized linear models
HCT: hematopoietic cell transplantation
IN: inpatient
MDS: myelodysplastic syndromes
MM: multiple myeloma
MPD: myeloproliferative disorders
NHL: non-Hodgkin’s lymphoma
OP: outpatient
PNH: paroxysmal nocturnal hemoglobinuria
SD: standard deviation
US: United States
