# Supplementary material for: Healthcare utilization and cost of cancer-related care prior to allogeneic hematopoietic cell transplantation for hematologic malignancies in the US: a retrospective real-world analysis
Source: BMC Health Serv Res. 2021 Oct 20;21:1125. doi: 10.1186/s12913-021-07150-4 (PMC8527718; doi:10.1186/s12913-021-07150-4)
Supplement: Supplementary file 1 — Additional file 1. [file 12913_2021_7150_MOESM1_ESM.docx]

**Supplementary Materials**

Supplementary Table 1: Demographic characteristics at index date of HCT patients in the Commercial Database

| **Demographics** | **All  Patients** | **ALL** | **AML** | **NHL** | **MDS** | **MPD** |
| --- | --- | --- | --- | --- | --- | --- |
|  | **N=2,667** | **N=346** | **N=984** | **N=447** | **N=330** | **N=148** |
| **Age (mean, SD)** | 50.1 (11.5) | 44.8 (13.4) | 50.4 (11.3) | 50.8 (9.9) | 54.7 (8.5) | 52.3 (10.3) |
| **Age group (N, %)** |  |  |  |  |  |  |
| 18-24 | 138 (5.2%) | 42 (12.1%) | 49 (5.0%) | 12 (2.7%) | 3 (0.9%) | 3 (2.0%) |
| 25-34 | 186 (7.0%) | 43 (12.4%) | 66 (6.7%) | 24 (5.4%) | 11 (3.3%) | 10 (6.8%) |
| 35-44 | 365 (13.7%) | 69 (19.9%) | 129 (13.1%) | 71 (15.9%) | 24 (7.3%) | 15 (10.1%) |
| 45-54 | 768 (28.8%) | 83 (24.0%) | 294 (29.9%) | 143 (32.0%) | 81 (24.6%) | 39 (26.4%) |
| 55-64 | 1,210 (45.4%) | 109 (31.5%) | 446 (45.3%) | 197 (44.1%) | 211 (63.9%) | 81 (54.7%) |
| **Male** | 1,470 (55.1%) | 185 (53.5%) | 484 (49.2%) | 288 (64.4%) | 194 (58.8%) | 75 (50.7%) |
| **Geographic region (N, %)** |  |  |  |  |  |  |
| Northeast | 523 (19.6%) | 78 (22.5%) | 196 (19.9%) | 80 (17.9%) | 68 (20.6%) | N.R. |
| North Central | 668 (25.1%) | 67 (19.4%) | 265 (26.9%) | 113 (25.3%) | 78 (23.6%) | N.R. |
| South | 988 (37.1%) | 126 (36.4%) | 342 (34.8%) | 168 (37.6%) | 122 (37.0%) | N.R.. |
| West | 476 (17.9%) | 74 (21.4%) | 175 (17.8%) | 84 (18.8%) | 60 (18.2%) | N.R. |
| Unknown | 12 (0.5%) | 1 (0.3%) | 6 (0.6%) | 2 (0.5%) | 2 (0.6%) | N.R. |
| **Insurance plan type (N, %)** |  |  |  |  |  |  |
| Comprehensive | 102 (3.8%) | 8 (2.3%) | 35 (3.6%) | 20 (4.5%) | 20 (6.1%) | 5 (3.4%) |
| EPO/PPO | 1,572 (58.9%) | 214 (61.9%) | 578 (58.7%) | 257 (57.5%) | 181 (54.9%) | 94 (63.5%) |
| POS/POS with capitation | 267 (10.0%) | 37 (10.7%) | 90 (9.2%) | 41 (9.2%) | 46 (13.9%) | 12 (8.1%) |
| HMO | 367 (13.8%) | 40 (11.6%) | 125 (12.7%) | 84 (18.8%) | 44 (13.3%) | 18 (12.2%) |
| CDHP/HDHP | 295 (11.1%) | 36 (10.4%) | 126 (12.8%) | 38 (8.5%) | 29 (8.8%) | 18 (12.2%) |
| Missing/unknown | 64 (2.4%) | 11 (3.2%) | 30 (3.1%) | 7 (1.6%) | 10 (3.0%) | 1 (0.7%) |
| **Urban** | 2,293 (86.0%) | 311 (89.9%) | 828 (84.2%) | 402 (89.9%) | 281 (85.2%) | 122 (82.4%) |
| **Year of index diagnosis^*^ (N, %)** |  |  |  |  |  |  |
| 2005-2009 | 627 (23.5%) | 62 (17.9%) | 171 (17.4%) | 163 (36.5%) | 58 (17.6%) | 40 (27.0%) |
| 2010-2013 | 1,232 (46.2%) | 161 (46.5%) | 495 (50.3%) | 194 (43.4%) | 158 (47.9%) | 60 (40.5%) |
| 2014-2017 | 808 (30.3%) | 123 (35.6%) | 318 (32.3%) | 90 (20.1%) | 114 (34.5%) | 48 (32.4%) |

n.r, not reported when >1 region has <30 patients.

^*^The index year distribution is at least partially attributable to changes over time in MarketScan data contributors and the number of covered lives in the database.

ALL, acute lymphocytic leukemia; AML, acute myelogenous leukemia; CDHP, consumer-driven health plans; HDHP, high-deductible health plan; EPO, exclusive provider organization; HCT, hematopoietic cell transplantation; HMO, health maintenance organization; MDS, myelodysplastic syndrome; MPD, myeloproliferative disorders; NHL, Non-Hodgkin’s lymphoma; POS, point of service plans; PPO, preferred provider organization.

Supplementary Table 2: Demographic characteristics at index date of HCT patients in the Medicare Database

| **MEDICARE PATIENTS** | | | | | | |
| --- | --- | --- | --- | --- | --- | --- |
| **Demographics^1^** | **All Medicare Patients** | **ALL*** | **AML** | **NHL*** | **MDS** | **MPD*** |
|  | **N=266** | **N=14** | **N=121** | **N=30** | **N=63** | **N=11** |
| **Age (mean, SD)** | 67.7 (3.4) | 68.0 (2.4) | 68.3 (3.7) | 66.0 (3.3) | 67.6 (2.9) | 66.5 (2.2) |
| **Age group (N, %)** |  |  |  |  |  |  |
| <65 | 17 (6.4%) | 0 (0.0%) | 6 (5.0%) | 3 (10.0%) | 4 (6.4%) | 0 (0.0%) |
| 65-69 | 181 (68.1%) | 10 (71.4%) | 76 (62.8%) | 24 (80.0%) | 43 (68.3%) | 10 (90.9%) |
| 70-74 | 62 (23.3%) | 4 (28.6%) | 34 (28.1%) | 3 (10.0%) | 15 (23.8%) | 1 (9.1%) |
| 75-79 | 5 (1.9%) | 0 (0.0%) | 4 (3.3%) | 0 (0.0%) | 1 (1.6%) | 0 (0.0%) |
| 80+ | 1 (0.4%) | 0 (0.0%) | 1 (0.8%) | 0 (0.0%) | 0 (0.0%) | 0 (0.0%) |
| **Male** | 163 (61.3%) | 11 (78.6%) | 65 (53.7%) | 20 (66.7%) | 43 (68.3%) | 6 (54.6%) |
| **Geographic region^2^ (N, %)** |  |  |  |  |  |  |
| Northeast | 55 (20.7%) | N.R. | N.R. | N.R. | N.R. | N.R. |
| North Central | 100 (37.6%) | N.R. | N.R. | N.R. | N.R. | N.R. |
| South | 67 (25.2%) | N.R. | N.R. | N.R. | N.R. | N.R. |
| West | 44 (16.5%) | N.R. | N.R. | N.R. | N.R. | N.R. |
| Unknown | 0 (0.0%) | N.R. | N.R. | N.R. | N.R. | N.R. |
| **Insurance plan type (N, %)** |  |  |  |  |  |  |
| Comprehensive | 98 (36.8%) | 6 (42.9%) | 41 (33.9%) | 9 (30.0%) | 24 (38.1%) | 5 (45.5%) |
| EPO/PPO | 134 (50.4%) | 6 (42.9%) | 64 (52.9%) | 19 (63.3%) | 30 (47.6%) | 5 (45.5%) |
| POS/POS with capitation | 10 (3.8%) | 1 (7.1%) | 6 (5.0%) | 0 (0.0%) | 1 (1.6%) | 1 (9.1%) |
| HMO | 22 (8.3%) | 1 (7.1%) | 10 (8.3%) | 2 (6.7%) | 6 (9.5%) | 0 (0.0%) |
| CDHP/HDHP | 1 (0.4%) | 0 (0.0%) | 0 (0.0%) | 0 (0.0%) | 1 (1.6%) | 0 (0.0%) |
| Missing/unknown | 1 (0.4%) | 0 (0.0%) | 0 (0.0%) | 0 (0.0%) | 1 (1.6%) | 0 (0.0%) |
| **Urban** | 225 (84.6%) | 13 (92.9%) | 100 (82.6%) | 27 (90.0%) | 56 (88.9%) | 7 (63.6%) |
| **Year of index diagnosis (N, %)** |  |  |  |  |  |  |
| 2005-2009 | 44 (16.5%) | 2 (0.1%) | 17 (14.1%) | 11 (36.7%) | 6 (9.5%) | 1 (0.1%) |
| 2010-2013 | 122 (45.9%) | 3 (21.4%) | 61 (50.4%) | 14 (46.7%) | 28 (44.4%) | 5 (45.5%) |
| 2014-2017 | 100 (37.6%) | 9 (64.3%) | 43 (35.5%) | 5 (16.7%) | 29 (46.0%) | 5 (45.5%) |

n.r, not reported when >1 region has <30 patients

^1^ Assessed on the index date; ^2^ Geographic regions are not reported for groups when >1 region has < 30 patients; *Results for cohorts with small sample sizes should be interpreted cautiously.

ALL, acute lymphocytic leukemia; AML, acute myelogenous leukemia; CDHP, consumer-driven health plans; HDHP, high-deductible health plan; EPO, exclusive provider organization; HCT, hematopoietic cell transplantation; HMO, health maintenance organization; MDS, myelodysplastic syndrome; MPD, myeloproliferative disorders; NHL, Non-Hodgkin’s lymphoma; POS, point of service plans; PPO, preferred provider organization.
